# Pipeline for the Rapid Development of Cytogenetic Markers Using Genomic Data of Related Species

**DOI:** 10.3390/genes10020113

**Published:** 2019-02-01

**Authors:** Pavel Kroupin, Victoria Kuznetsova, Dmitry Romanov, Alina Kocheshkova, Gennady Karlov, Thi Xuan Dang, Thi Mai L. Khuat, Ilya Kirov, Oleg Alexandrov, Alexander Polkhovskiy, Olga Razumova, Mikhail Divashuk

**Affiliations:** 1 Laboratory of Applied Genomics and Crop Breeding, All-Russia Research Institute of Agricultural Biotechnology, Timiryazevskaya str. 42, Moscow 127550, Russia; pavelkroupin1985@gmail.com (P.K.); vika-kuz367@yandex.ru (V.K.); akabos1987@gmail.com (D.R.); karlovg@gmail.com (G.K.); razumovao@gmail.com (O.R.); 2 Center of Molecular Biotechnology, Russian State Agrarian University-Moscow Timiryazev Agricultural Academy, Timiryazevskaya str. 49, Moscow 127550, Russia; alina.korotaeva@gmail.com (A.K.); xuxu.dang91@gmail.com (T.X.D.); hoamoclantt_36@yahoo.com (T.M.L.K.); polkhovsky.a.w@gmail.com (A.P.); 3 Laboratory of Marker-Assisted and Genomic Selection of Plants, All-Russia Research Institute of Agricultural Biotechnology, Timiryazevskaya str. 42, Moscow 127550, Russia; kirovez@gmail.com; 4 Laboratory of Plant Cell Engineering, All-Russia Research Institute of Agricultural Biotechnology, Timiryazevskaya str. 42, Moscow 127550, Russia; olegsandrov@gmail.com

**Keywords:** DNA repeats, satellite DNA, tandem repeats, bioinformatics search, whole-genome sequencing, cytogenetic markers, fluorescence in situ hybridization, wheat, rye, triticale

## Abstract

Repetitive DNA including tandem repeats (TRs) is a significant part of most eukaryotic genomes. TRs include rapidly evolving satellite DNA (satDNA) that can be shared by closely related species, their abundance may be associated with evolutionary divergence, and they have been widely used for chromosome karyotyping using fluorescence in situ hybridization (FISH). The recent progress in the development of whole-genome sequencing and bioinformatics tools enables rapid and cost-effective searches for TRs including satDNA that can be converted into molecular cytogenetic markers. In the case of closely related taxa, the genome sequence of one species (*donor*) can be used as a base for the development of chromosome markers for related species or genomes (*target*). Here, we present a pipeline for rapid and high-throughput screening for new satDNA TRs in whole-genome sequencing of the *donor* genome and the development of chromosome markers based on them that can be applied in the *target* genome. One of the main peculiarities of the developed pipeline is that preliminary estimation of TR abundance using qPCR and ranking found TRs according to their copy number in the *target* genome; it facilitates the selection of the most prospective (most abundant) TRs that can be converted into cytogenetic markers. Another feature of our pipeline is the probe preparation for FISH using PCR with primers designed on the aligned TR unit sequences and the genomic DNA of a *target* species as a template that enables amplification of a whole pool of monomers inherent in the chromosomes of the *target* species. We demonstrate the efficiency of the developed pipeline by the example of FISH probes developed for A, B, and R subgenome chromosomes of hexaploid triticale (BBAARR) based on a bioinformatics analysis of the D genome of *Aegilops tauschii* (DD) whole-genome sequence. Our pipeline can be used to develop chromosome markers in closely related species for comparative cytogenetics in evolutionary and breeding studies.

## 1. Introduction

A large proportion of eukaryotic genomes are represented by various classes of repetitive DNA sequences that are mainly dispersed (mainly transposons and retrotransposons) and arranged in tandem (ribosomal RNA, protein-coding gene families, satellites, telomeric DNA, and centromeric DNA). Highly abundant tandem repeat (TR) DNA sequences, known as satellites, form a significant part of many eukaryotic genomes and are classified as microsatellite, satellite (satDNA), and ribosomal DNA genes. SatDNAs are organized in large tandem arrays (tens of kilobases up to megabases) of highly repetitive noncoding monomers with an average length of 100 to 700 bp organized in head-to-tail fashion. SatDNA is usually found in heterochromatin and may form centromeres and telomeres of chromosomes; in higher plants, it is also located in interstitial chromosome regions. SatDNA may play a structural role in genome organization and in regulation of gene expression by epigenetic modifications; variations in satellite abundance have been associated with differences and genetic incompatibilities between species. In plants, satDNA can comprise from 0.1% up to 36% of the genome; in wheat and rye, for example, it occupies up to 5% and 12%, respectively [1,2,3]. Amplification of the monomers, their homogenization, and their changes occur via mechanisms of concerted evolution, including slip replication, mutations, crossing over, and others. One satDNA family may be present in one species, and different species may share a common set or have species-specific sets. That makes it possible to use the same satDNA family as a tool to compare different closely related species and their hybrids. Different satDNA families are fast evolving, and due to their high evolutionary rate, they are species- or genus-specific [4,5,6]. Therefore, identifying satDNA in phylogenetically distant species is not possible; inversely, once found in one genome, a given repeat may be found in a closely related genome.

A high abundance of satDNA in the genome makes it easily accessible, and a number of strategies to search for and isolate TRs have been designed: (a) isolation of satDNA by restriction of endonuclease digestion; (b) cutting out relic DNA of the gel with subsequent digestion, cloning, and colony filter hybridization; (c) amplification with specific primers; (d) colony filter hybridization; and (e) genomic self-amplification (“self-priming”) [1,3]. Recent advances in whole-genome next-generation sequencing and bioinformatics provide another way to rapidly detect satDNAs that are applicable as fluorescence in situ hybridization (FISH) markers at a reasonable cost. As an alternative to experimental approaches, computational bioinformatics tools can be used to identify new repetitive DNA, including satDNA, in genomic sequence data. Earlier algorithms such as Tandem Repeats Finder [7] required long input sequences, and were followed by more advanced bioinformatics toolkits that can handle even shotgun low-coverage sequencing (“genome skimming” data) such as RepeatExplorer [8], satMiner, TAREAN [9], and pyTanFinder [10]. Applying different tools in a complex may help to accomplish the following tasks: (i) identify repetitive DNA, (ii) assign to a repetitive family, and (iii) quantify genomic abundance and/or sequence variation [1,3].

Since high-copy-number satDNAs form long arrays in the chromosome locus, they are well-detected using a FISH assay when a labeled monomer or its fragment is used as a probe. The isolated satDNA is used to develop molecular cytogenetic markers, which have been widely and successfully applied in comparative cytogenetic evolutionary and breeding studies [4,5,6]. Therefore, a rapid and high-throughput assay for the de novo identification of satDNAs and verification of their feasibility as FISH markers is necessary to saturate FISH maps for fine cytogenetic studies. 

The approaches for rapid and cost-efficient whole-genome sequencing are rapidly developing, hence the availability of new satDNAs that can be used as cytogenetic markers is increasing. Although in recent research, novel cytogenetic markers have been designed on the basis of next generation sequencing (NGS) data of one species (designated here as *donor* genomes or species) and applied in the genomes of closely related species (designated as *target* genomes or species) [11,12,13], the methodological aspect of this problem per se has not yet been considered. In this paper, we present a pipeline for rapid and high-throughput screening of new satDNA TRs in the whole-genome sequencing data of *donor* genome and the development of cytogenetic markers based on them that can be applied in the *target* genome. The proposed pipeline is a system of procedures that logically follow one after the other. The backbone of our pipeline consists of the following steps: in silico screening of NGS data for satDNA, primer design for TR monomers, conventional PCR, quantitative PCR, and FISH with PCR-derived or oligonucleotide probes on the chromosomes of *donor* and *target* species. The novelty of our pipeline is the following: For the first time, we propose to apply a qPCR assay that combines both qualitative and quantitative assessment of a particular TR in related genomes; this facilitates the search for common, highly abundant satDNAs that can be converted into molecular cytogenetic markers. Additionally, we propose that PCR for probe synthesis should be performed using not only DNA templates of the *donor* species but also DNA of the related *target* species; this may strengthen the hybridization of the probe on the chromosomes of the *target* genome due its higher affinity to the DNA of *target* species. 

The efficiency of the presented pipeline was demonstrated in the experiments. The *Aegilops taushii* genome (contributor of the D subgenome) was chosen for the search of TRs as a *donor* species, as it has been sequenced and Triticeae species have large, well-characterized chromosomes. Hexaploid triticale (BBAARR, 2n = 2x = 42) and *Triticum aestivum* (BBAADD, 2n = 2x = 42) were used as *target* species for amplification of the TR’s monomers and in situ hybridization on the chromosome preparations. Hexaploid triticale and wheat were used as objects because they both contain A and B subgenomes from wheat, which enables studying the D genome TRs in the closely related genomes; additionally, triticale contains subgenome R from rye *Secale cereale*, which enables studying the D genome TRs in the distantly related genome, and *Triticum aestivum* contains a D subgenome, which enables studying the D genome TRs in the D subgenome itself. Using polyploid species provides an opportunity for simultaneous comparison of the signal intensity of FISH on the chromosomes of different subgenomes; the comparison between chromosome plates of different species (genomes) on different preparations would not be correct due to variations in hybridization and stringency washing conditions between slides. Additionally, it reduces the number of experiments as compared to the separate studies of A, B, D, and R subgenome donors.

## 2. Materials and Methods 

### 2.1. Plant Material

*Triticum monococcum* subsp. *aegilopoides* (CItr 17665), *Ae. speltoides* (PI 487235), and *Ae. tauschii* (CIae 3) were provided by the Germplasm Research International Network (Washington DC, USA); *T. aestivum* cv. Ivolga and hexaploid triticale cv. Solovey Kharkovskiy were provided by the Field Experimental Station of the Russian State Agrarian University (Moscow, Russia); *T. durum* cv. LD222 and *S. cereale* cv. EM1 were provided by N.I. Vavilov Research Institute of Plant Industry (Saint Petersburg, Russia).

### 2.2. Bioinformatics Analysis

The *Ae. taushi* genome contigs available at National Center for Biotechnology Information NCBI (MCGU01000001–MCGU01068537) were used for the TR search using Tandem Repeats Finder (TRF) software Version 4.09 (Boston University, Boston, MA, USA) [7] and pyTanFinder (https://github.com/Kirovez/pyTanFinder) [10], setting the minimum length of TRs at 5 bp and the minimum local copy number (number of copies in the contig) at 5, followed by the generation of a FASTA file with selected sequences. Then TR sequences were clustered by the CD-HIT tool [14] and total abundance (TA) for each selected TR per cluster was calculated.

TRF resulted in 1,403,751 sequences, 49,019 of them with monomer length >5 bp and minimum local copy number >5. CD-HIT and BLASTn-based clustering further reduced the number of TR sequences to 8649 and 2019, respectively. The final set comprised TRs with a maximum TA value of 8,785,741 bp.

### 2.3. PCR

DNA was extracted from the seedlings according to the protocol in Bernatzky and Tanksley (1986) [15]. 

On the basis of the monomers for each TR, the primers were designed using Primer 3.0 v 4.1.0 (http://primer3.ut.ee) (Table 1). PCR was conducted in a Tetrad 2 Thermal Cycler (Biorad, Hercules, CA, USA) in a total volume of 25 μL containing 2.5 μL 10× buffer, 2 μM dNTP, 1 unit Taq DNA polymerase, 1 μL primer (10 pM/μL), and 80–100 ng total genomic DNA. Each PCR amplification was performed under the following conditions: 95 °C for 15 min, followed by 35 cycles of 95 °C for 30 s, 60 °C for 45 s, and 72 °C for 30 s, and a final extension at 72 °C for 10 s. The PCR products were fractionated on 1% agarose gel in Tris-borate-EDTA (TBE) buffer under 6 V/cm.

### 2.4. Real-Time PCR

Real-time quantitative PCR (qPCR) was performed as in Yaakov et al. (2013) [16] to relatively quantify the following 10 TRs in the genomes of *T. aestivum*, *T. durum*, and *S. cereale*: P720, P332, P631, P170, P496, P525, P431, P317, P699, and P497. Each experiment was repeated independently at least three times. In each experiment, the qPCR amplification was run in three technical replicates on the LightCycler® 96 instrument (Roche Diagnostics, Mannheim, Germany). Each reaction was performed in a 15 μL volume consisting of 2.5 μL of reaction mix containing Eva Green® (Syntol LTD, Moscow, Russia), serially diluted DNA template (10, 2, 0.4, and 0.08 ng), and 1.0 μL each of forward and reverse primer (10 pM/μL, Table 1). As a reference gene, we used a single-copy *VRN1* gene as described in Yaakov et al. (2013) [16].

The relative quantity of each TR was calculated based on the primer efficiency and threshold cycles according to the following formula [17]:

$$Relative\;quantity=E^{\left(Cq\left(ref\right)-Cq\left(TR\right)\right)}$$

where Cq(ref) stands for a threshold cycle of the reference gene (*VRN1*) and Cq(TR) stands for a threshold cycle of a particular TR; the efficiency of the PCR reactions (E) was determined using a standard curve through serial dilutions for each primer pair for each species and calculated using the LightCycler® (Roche Molecular Systems Inc., Pleasanton, CA, USA) 96 software (see details in Supplementary Table S1 and Figures S1–S6). In each species, the relative quantity of each TR (RQ) compared to the reference gene and the standard deviations were calculated using the LightCycler^®^ 96 software.

### 2.5. Fluorescence In Situ Hybridization (FISH)

The physical localization of the 10 TRs was analyzed using the FISH procedure. A chromosome spread preparation was made from root tip as described in Kroupin et al. (2011) [18]. Good slides with well-spread chromosomes were stored at 4 °C until use. FISH was carried out following the procedure in Divashuk et al. (2016) [19] using three variants of probes: (i) PCR products produced using the TRs’ primers and *T. aestivum* (ABD probe), *T. durum* (AB probe), and *S. cereale* (R probe) DNA as a template, and subsequently labeled using biotin- or digoxigenin (DIG)-PCR labeling mix (Roche Molecular Biochemicals); (ii) biotinylated synthetic oligonucleotides oligo332-1 (bio-CGA GTG AGA GGA TTG CTC TTC ACT CGG TAG ATT TTT), oligo332-2 (bio-AAA ACT TAG GCG AGT ACG GGA CTG CAG CTA AGC CCC), and oligo332-3 (bio-TTG CCC TTC ACT CGG TAG GAT TTT TCA AAC TA) (Syntol LTD); and (iii) fluorescein amidite (FAM)-labeled synthetic oligonucleotides for rye-specific probes pSc200 and pSc250 (Syntol LTD). After hybridization, the chromosomes were counterstained with 1 mg/ml 4′,6-diamidino-2-phenylindole (DAPI). The detection was performed for probes (i) and (ii) using streptavidin-conjugated Cy3 or FITC (Roche); signals in all variants were visualized using an AxioZeiss Imager V1 (Carl-Zeiss, Oberkochen, Germany) fluorescence microscope with Cy3 filter. The results were recorded with an AxioCam Mrm Zeiss camera (Carl-Zeiss, Oberkochen, Germany) and contrasted using AxioVision. 

## 3. Results

### 3.1. The Pipeline

Our pipeline is an assay system for rapid and high-throughput selection of new satDNA sequences using the whole-genome sequence of a species (*donor*) and the development of chromosome markers based on them to be applied in the study of a related species (*target*). The proposed system is a coherent sequence of assays that logically follow one after the other. The backbone of the pipeline consists of the following steps: in silico screening of NGS data for satDNA, primer design for TR monomers, conventional PCR, quantitative PCR, and FISH with PCR-derived or oligonucleotide probes on the chromosomes of *donor* and *target* species.

- **Step 1:** Identify TRs including satDNA in the genomic sequence of the *donor* species using bioinformatics tools such as RepeatExplorer (http://repeatexplorer.org), TAREAN (http://repeatexplorer.org), Tandem Repeats Finder (https://tandem.bu.edu), pyTanFinder (https://github.com/Kirovez/pyTanFinder), and other pipelines. The resulting output data is the set of TR units and their characteristics, such as sequence of repeat unit, length of clusters, and copy number in the *donor* genome; the most promising repeats can be selected in this step and used in Step 2.
- **Step 2:** Design primer based on the sequence alignment of the selected TRs units using Primer3 (http://primer3.ut.ee), Oligo (https://www.oligo.net), and other software. When necessary, some alternative primer sets may be designed, for example, if base content in the repeat monomer sequence does not allow for satisfactory primers to be designed. The result of Step 2 is the primers that can be used for repeat unit PCR amplification. If no efficient primers can be designed, the labeled synthetic oligonucleotide probes are used instead of PCR amplicons in further steps.
- **Step 3:** Validate the designed primers by PCR using genomic DNA of the *donor* and *target* species as a template. If the primers fail to amplify a particular TR monomer, then another pair of primers should be developed. In case all designed primers for a given repeat unit result in amplification failure, for the FISH experiments, a labeled synthetic oligonucleotide should be used for this repeat as a probe, or it should be excluded from the experiment.
- **Step 4:** Quantify the identified TRs using qPCR with the designed and validated primers and the DNA of the *target* and *donor* species (genomes) as a template; a single-copy gene is used as a reference to normalize the fluorescence level corresponding to the abundance of TRs relative to an endogenous control. This step is one of the most crucial, as it allows ranging the selected repeats corresponding to their copy number in the genome. On the one hand, the most abundant repeats are the most likely candidates for cytogenetic markers. On the other hand, the TRs that demonstrate absence or extremely low abundance in the *target* species can potentially be used in a species-specific manner for the *donor* species. In the experimental section of this work, repeats with different abundances were compared to illustrate this proposition.
- **Step 5:** Synthesize the labeled probes and FISH experiments. The efficiency of the developed chromosome markers is validated directly via hybridization on the chromosomes of the *target* species or hybrids/amphidiploids containing both *target* and *donor* genomes; FISH in the *donor* species may be necessary as a control. Both labeled oligonucleotides and labeled PCR amplicons may be used as probes; however, the latter is more advantageous since the product of amplification is heterogeneous and contains a pool of sequences that are the same/similar in their primer binding sites and may be different in the inner part. Additionally, application of the labeled PCR product enables selection of the most appropriate primer pairs in case several primer pairs have been designed.

The efficiency of the presented pipeline was demonstrated in a series of experiments on the model of research on the genomes of cereals. As a *donor* we used the genome D (species: *Ae. taushii* (DD, 2n = 2x = 14)), and as *targets*, genomes A, B, R (species: hexaploid triticale (BBAARR, 2n = 2x = 42), and *T. aestivum* (BBAADD, 2n = 2x = 42).

### 3.2. Steps 1 and 2: Bioinformatics Analysis of Ae. taushii Genome, Search for TRs, and Primer Design

To verify our pipeline, we applied it to *Ae. taushii* genome contigs available at NCBI (MCGU01000001–MCGU01068537). Tandem Repeats Finder (TRF) resulted in 1,403,751 sequences, 49,019 of them with monomer length >5 bp and minimum local copy number >5. CD-HIT and BLASTn-based clustering further reduced the number of TR sequences to 8649 and 2019, respectively. The final set comprised TRs with maximum total abundance (TA) value 8,785,741 bp. 

To further prove that the calculated TA values correlated with real genome occupancy using the TRs, we selected with different copy numbers of the monomer 10 TRs: of them, 4 (P720 and P317 (TA:8785742), P170 (TA:5103712), and P332 (TA:2553650)) had a high TA value (2.5–8.5 Mb); 2 (P631 (TA:856796) and P699 (TA:672871)) had medium TA (600–900 Kb); and 4 (P496 (TA:91576), P525 (TA: 32978), P431 (TA: 20406), and P497(TA:11223)) had low TA (20–100 Kb). 

The repeats with different copy numbers of monomers in the *Ae. taushii* genome were selected for the following studies according to the presented pipeline. The primers for amplification of the selected TRs were designed based on the sequence alignment of the TRs’ units (Table 1).

### 3.3. Steps 3 and 4: PCR and qPCR Analysis of TRs

**Figure 1:**
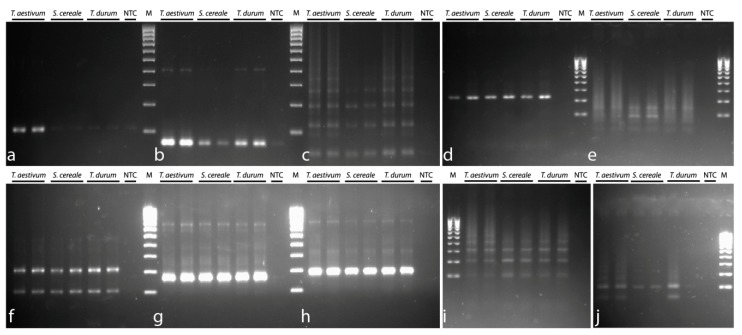
Electrophoresis of primers for monomers: (**a**) P170, (**b**) P317, (**c**) P332, (**d**) P720, (**e**) P496, (**f**) P431, (**g**) P525, (**h**) P631, (**i**) P497, and (**j**) P699. M—size standard (100 bp DNA ladder).

PCR experiments with the designed primers were conducted using three DNA templates, *T. aestivum*, *T. durum*, and *S. cereale*. Fragments of the expected size were amplified in all TRs. In P332, P631, P496, P525, P431, P317, P699, and P497, there were some additional fragments whose size was twofold (or more) larger than a single TR unit. In P720 and P170, only fragments with the expected size were detected (Figure 1).

The successful PCR results made it possible to conduct a comparative assessment of their copy numbers in the studied genomes using qPCR. For a *donor* genome, in this case *Ae. taushii*, we could easily assess the copy number and potential suitability of TRs to create cytogenetic markers according to the bioinformatics data of the genome using various software. However, in the case of *target* genomes, we do not have such tools. The standard PCR procedure simply shows us the presence of amplification of primers for a TR, but not its copy number in the *target* genome. Therefore, at this stage, qPCR is a very useful tool for the *target* genomes. This step is required to rank the studied repeats in their abundance and identify the most promising TRs for FISH localization at the *target* genome, i.e., high-copy-number TRs. The results of TR quantification using qPCR in relation to a single-copy gene *VRN1* in the genomes of *T. monococcum* (A^m^A^m^), *Ae. speltoides* (SS, candidate donor for B genome), *Ae. tauschii* (DD), *T. durum* (BBAA), *T. aestivum* (BBAADD), and *S. cereale* (RR) are depicted in Supplementary Figures S1–S6.

For *Ae. taushii* (DD) and *T. aestivum* (BBAADD), taking into account the resolution of the qPCR method (which depends on the size of the PCR product, GC content, reaction efficiency, etc.), the ranking of replicas on the copy number inside the genomes is similar to the data on the bioinformatics analysis of the *Ae. taushii* genome, with the exception of repeat P170. The most high-copy qPCR method was for TRs P317, P720, and P332; the rest showed significantly lower copy numbers (Supplemental Figures S3 and S5). 

When analyzing *target* species by the qPCR method, *T. durum* (BBAA), and separately the donors of subgenomes A (*T. monococcum* (A^m^A^m^)) and B (*Ae. speltoides* (SS)), P332 was the most highly copied and promising. P496, P720, P631, P497, P431, and P525 had lower copy numbers. TRs P317 showed the absence or very low copy numbers in genomes A and B, therefore no potential for creating cytogenetic markers for those genomes (Supplemental Figures S1, S2, and S4). When analyzing these data, it should be taken into account that *Ae. speltoides* carries the genome S, related to the genome B, but rather different from it. 

When analyzing *target* species uisng the qPCR method, rye (RR) was the most highly copied with P496, P431, and P525 (Supplemental Figure S6). TRs P317, P170, and P332, the most highly copied ones in *Ae. taushii*, showed a complete absence of the R genome, and therefore no potential for the development of cytogenetic markers for that genome.

### 3.4. Steps 5 and 6: Probe Synthesis and FISH Experiments

In this work, two variants of probes were used: (i) a PCR product labeled with modified nucleotides; and (ii) biotinylated synthetic oligonucleotides (oligo-FISH probes).

The first variant was amplified using PCR with different templates. In triticale FISH experiments, two types of the labeled PCR product were used as a probe (except for P170): the first type was obtained using PCR with the *T. durum* (BBAA) genomic DNA as a template (AB probe), while the second type was prepared using the *S. cereale* (RR) genomic DNA as a template (R probe). For P170, neither AB nor R probes were synthesized, as the amplification from the corresponding templates failed. In *T. aestivum* FISH experiments, the probe was synthesized using PCR with the *T. aestivum* genomic DNA as a template with subsequent labeling (ABD probe). The second variant of the probe was represented by oligo-FISH probes P332 and P496 (Supplementary Table S2).

**Figure 2:**
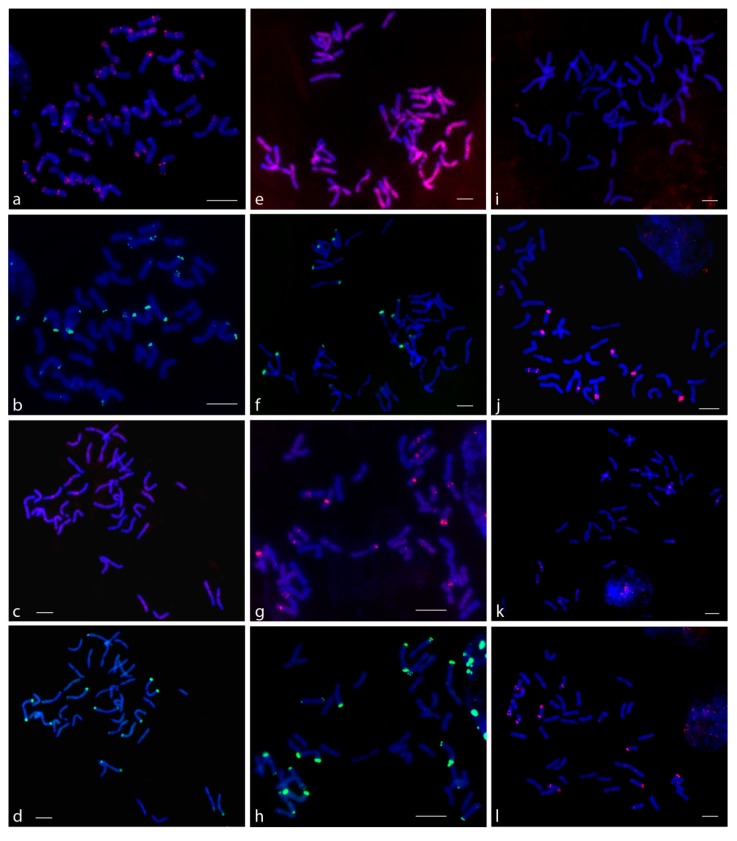
Hybridization of probes on chromosomes of triticale (BBAARR) and *T. aestivum* (BBAADD). Sequential FISH with TR probe and with oligo-probe pSc200 and pSc250 for detection of chromosome genome R on the root-tip cells at mitotic metaphase in triticale (BBAARR): (**a**) P720 (AB), (**b**) same cell with pSc200 and pSc250 (green), (**c**) P631 (AB), (**d**) same cell with pSc200 and pSc250 (green), (**e**) P496 (oligo), (**f**) same cell with pSc200 and pSc250 (green), (**g**) P332 (AB), and (**h**) same cell with pSc200 and pSc250 (green). FISH with TR probe on root-tip cells at mitotic metaphase *T. aestivum* (BBAADD): (**i**) P525 (ABD), (**j**) P317 (ABD), (**k**) P431 (ABD), and (**l**) P170 (ABD). Chromosomes counterstained with DAPI (blue). Scale bar = 10 μm.

The FISH experiments with P720 showed that signals were localized to all chromosomes of subgenomes A and B in the subtelomeric region (but with varying degrees of intensity signals) and on several pairs of chromosomes in the pericentromeric region (Figure 2). In *T. aestivum*, signals on the 14 chromosomes in the subtelomeric region were more intense than on the other chromosomes. When comparing data on *T. aestivum* and triticale, these 14 chromosomes were assigned to subgenome D (data not shown). In the variant with triticale, the signals of the AB and R subgenome probes corresponded on the same metaphase plates and signals derived from different subgenomes co-localized together. This suggests that TR P720 is rather conservative, being included in the nucleotide sequences of its monomers. The subsequent FISH on the same metaphase plate using rye-specific pSc200 and pSc250 probes revealed that P720 was localized in the pericentromeric region of two pairs rye (R subgenome) chromosomes. 

For P332, only the ABD and AB PCR-labeled probes were synthesized. As the amplification of R probe with genomic DNA of rye as a template did not succeed, we used three oligo-FISH probes on a different part of the P332 instead. The results of FISH experiments with *T. aestivum* chromosome plates demonstrated that the signals of the ABD probe and the oligo-FISH probes corresponded to each other. However, when the ABD PCR-derived probe was applied, a number of nonspecific and nonreproducible signals and a rather strong nonspecific background were observed. The same results were obtained when FISH experiments on triticale chromosomes with the AB PCR-derived probe were compared to those with oligo-FISH probes. The number of observed signals and the sequential FISH with pSc200 and pSc250 rye-specific probes indicated that the P332 probes hybridized to chromosomes of subgenomes A and B (in total, on 7 pairs of chromosomes). Additionally, minor signals were detected in the centromeric region and short arm of one pair and in the centromeric region and the middle of the short and long arms of another pair of R subgenome chromosomes.

The P317 probe successfully hybridized only to the D subgenome chromosomes. The FISH signals were bright and clear in chromosomes. When FISH with the P317 and rye-specific pSc200 and pSc250 probes was conducted on triticale metaphase plates, the P317 signals were not detected on the R subgenome chromosomes. The same results were observed in both cases when the AB and R probes were used. We used the R probe amplified from the genomic DNA of rye to find out if the rye-specific variants of P317 were localized to R subgenome chromosomes, but the lack of P317 signals on rye chromosomes in triticale proved that this repeat did not form large tandem clusters that could be visualized by FISH.

The other three TRs (P431, P496, and P525), which were selected as low-copy TRs based on bioinformatics processing of the *Ae. taushii* genome and the second priority TRs based on qPCR, did not show clear signals using FISH. Their dispersed signals were detected on the chromosomes of subgenomes A, B, and R without any specificity for any of the genomes. However, some interesting results were obtained with P170 and P631. PCR with the P170 primers succeeded only when the template genomic DNA of *T. aestivum* was used and failed in cases of *S. cereale* and *T. durum* genomic DNA. In FISH experiments with triticale, when the ABD probe was used, P170 signals were not detected. Some signals were observed in FISH experiments on *T. aestivum* chromosomes only. Therefore, results of amplification and in situ hybridization allow us to suggest that P170 is presumably a D subgenome-specific repeat. As for P631, most chromosomes of different subgenomes had dispersed signals, but two triticale and three *T. aestivum* chromosome pairs were distinguished from the others by large pericentromeric P631 signals. The subsequent hybridization with the rye-specific pSc200 and pSc250 probes on the same triticale metaphase plate showed that one of the large P631 signals was localized to the R subgenome chromosome pair.

P497 and P699 were successfully amplified, but when FISH was performed, they did not show signals on either wheat or triticale chromosomes.

## 4. Discussion

The development of our pipeline was based on two priorities, the rapidity of cytogenetic marker development and the efficiency of developed markers. Consequently, the first step is to choose the part of the genome that would satisfy these criteria. As most authors have reported, TRs and bacterial artificial chromosome (BAC) clones are often used as cytogenetic markers. Repeated DNA occupies a large chromosome region and can be easily detected using FISH. The cytogenetic markers based on TRs are widely used for comparative cytogenetics to study chromosome collinearity and to identify chromosomal rearrangements in evolutionary and breeding studies [5,6]. Although in some cases, BAC clones can be efficient as probes as well, their preparation is rather complex. On the other hand, cytogenetic marker development using TRs can be both effective and rapid. Thus, the TR fraction of the genome was chosen as the basis for the pipeline development. 

The next issue is to choose approaches and tools for the rapid search for and selection of suitable DNA repeats. Until recently, the search for DNA repeats was conducted using different procedures, such as separation of DNA fractions in the density gradient of cesium chloride, digestion of total DNA by restriction enzymes and band cutting from the gel, self-priming and cloning, etc. However, these techniques are time- and labor-consuming. At the same time, bioinformatics screening of the whole-genome sequence using effective software tools significantly accelerates and facilitates the search for novel TRs. The whole-genome sequence of a given species can be used in the in silico search for TRs, but such an approach requires the availability of good-quality reads [20]. Recent research used an alternative strategy based on NGS-based repeat findings in one species (*donor*) with consequent in situ hybridization on chromosomes of its related species (*target*). 

As a rule, the number of output TRs is rather large. Therefore, the next question is how to select the most promising repeats found in the *donor* genome to rapidly develop cytogenetic markers for *target* species. First, they can be estimated in silico based on the parameters of the selected TRs. It was reported that sequences larger than 10 kb can be successfully visualized on chromosomes using in situ hybridization [21]. If the target sequence is represented by a repeat, then the length of this array depends on the number of tandemly arranged monomers and the monomer length. The most prospective repeats must have a maximum value of these parameters. In this work, the repeats with different parameter values were selected for FISH experiments. As the results demonstrated, the repeats with high values (P332, P720, P170, and P631) had bright clear FISH signals and were successfully localized to the chromosomes.

Then, the prospective for the transformation of the selected TRs into cytogenetic markers can be estimated experimentally. It can be estimated directly using the FISH procedure, but since it is time- and labor-consuming, TR abundance should be estimated preliminarily, as in silico analysis does not guarantee that the newly found TR probes will be hybridized to the chromosomes, even of the *donor* species. In several papers, the abundance of TRs in related genomes was compared using Southern hybridization [22,23], which is not a high-throughput technique and requires radioactive labels [24,25]; to verify the presence (not the abundance) of TRs in the related genome, conventional PCR is also used [26,27]. We propose that a qPCR assay be applied as a tool to preliminarily estimate the abundance of TRs in the *target* species. qPCR combines qualitative and quantitative assessment of TR presence in related genomes and has been shown to be an efficient tool to analyze DNA repeat abundance in a genome [16]. Unlike in situ and Southern hybridization, real-time qPCR enables quick, easy, and cheap copy-number estimation of newly found TRs; unlike in silico analysis, it does not require the whole-genome sequence of the *target* species to evaluate TR copy numbers. qPCR makes it possible to select for the TRs that could be either common or specific to related genomes. 

In our experiments, P317, P720, and P332 were in the top list of repeats as a result of qPCR testing with the genomic DNA of *T. aestivum* as a template. Consequently, the repeats with maximum values of such parameters as unit length and number of tandemly arranged monomers and high abundance according to qPCR test should be used in FISH experiments first. In addition, it is equally important to eliminate TR high copies for the *donor* species, but low copy or absent in the *target* species. A good illustration is P317.

The next important issue is the proper technique of probe preparation. In recent papers, comparative cytogenetic analysis was performed mainly using the limited set of well-known reliable conservative TR probes, including ribosomal DNA probes, which requires the availability of BAC clones or plasmids [5,6,23,28,29,30]. Komuro et al. (2013) [31] revealed that 47 FISH-positive repeats constructed the genomic library of wheat and examined 2000 plasmids for signals using FISH. As an alternative to plasmids probes, synthesized non-SSR oligonucleotides were proposed and then applied in subsequent studies [20]. In other studies, PCR with the primers for TR monomers were used to prepare FISH probes using the genomic DNA of the *donor* species as a template, and then these probes were used for FISH on the chromosomes of related (*target*) species [11,12,28,29,30,32]. In our pipeline, we used PCR with the primers designed on the aligned monomer sequences, as it would more probably result in a population (pool) of different PCR products that are the same only in the flanking region, even when amplified from the same DNA template; unlike homogeneous oligonucleotides or plasmid clones, it may produce a more saturated FISH pattern on chromosomes. We propose that PCR should be performed with TR primers using the DNA template of not only the *donor* species but also the related *target* species. It may increase the chances for PCR probe hybridization to chromosomes of related species, since the inner region of the PCR-derived probe has higher affinity to the *target* species genome. For the probe preparation and qPCR assay, the same primers can be applied. 

A progressive single-gene FISH map approach was developed by Danilova et al. (2014) [24] and successfully applied in several studies on the relationships and chromosomal rearrangements within Triticeae [24]. Such an approach also can be used for in silico searches for novel cytogenetic markers on the basis of a DNA sequence database; however, this advantageous technique requires the availability of a cDNA clone library. Another type of cytogenetic marker widely used for comparative cytogenetic studies is microsatellites [24,28,32]. However, their transfer from related species cannot be accompanied by real-time qPCR estimation and their presence and abundance can only be estimated using PCR or directly by FISH.

As the selected high-copy TRs were successfully converted into cytogenetic markers, we can state that the final goal of our pipeline, i.e., rapid development of new cytogenetic markers for a *target* species based on the whole-genome sequence of the *donor* species, has been achieved. Different FISH experiments showed that the D subgenome TRs were localized to A, B, and R subgenome chromosomes. It is interesting that most of the selected repeats in the pipeline (P720, P332, P631, and P317) are highly homologous to the previously published repeats used as cytogenetic markers [31].

Thus, the presented pipeline is a powerful tool for developing new, efficient cytogenetic markers in closely related species using the whole-genome sequence of one of them.

## Figures and Tables

**Table 1:** Designed primers for TR monomer amplification and their characteristics.

Repeat Name	Primers	Annealing Temperature (°C)	Product Size (bp)
P720	F: 5’-AGCCACGTCATCAACTTTCA-3’	60	222
	R: 5’-TGTCCAGTTTGTACGCGAAG-3’		
P332^*^	F: 5’-GCTCTTCACTCGGTAGGATTT-3’	58	44
	R: 5’-TCCCGTACTCGCCTAAGT-3’		
P631	F: 5’-TGACACCATGCCAAGATTCA-3	60	191
	R: 5’-CCAGTGCACCAAGGTTGTTT-3’		
P170	F: 5’-TCCTTGGAAGAATCTAGTCGTCA-3’	60	106
	R: 5’-TCGGTTTTGCGCAGTGTTAA-3’		
P496	F: 5’-CTGGAGAGGGCTGGATGA-3’	58	51
	R: 5’-TCCACCGGCTACTGTTCA-3’		
P525	F: 5’-AATGAACCCGGAAAAGTGCC-3’	60	160
	R: 5’-TCGAACCATCACCGAGGAAG-3’		
P431	F: 5’-AGCATGCTTGACAACTTGGA-3’	60	216
	R: 5’-TGTTATCTTGTGCAATGTGGTG-3’		
P317	F: 5’-AGTCATTTGAACTCCAGCCTATT-3’	59	97
	R: 5’-GGAAATTGATGACGTCGCTTTG-3’		
P699	F: 5’-GTGTGTGTGTGTGTCGAGG-3’	60	102
	R: 5’-ATGGTCGGGTTGCAACTG-3’		
P497	F: 5’-CCGACTACAAATGGCCCAAC-3’	59	100
	R: 5’-GGCCTTTTAGCGACCCATTT-3’		

* Oligonucleotide probes were additionally used for this TR: oligo332-1, oligo332-2, and oligo332-3.

## Supplementary Materials

The following are available online at http://www.mdpi.com/2073-4425/10/2/113/s1: Figure S1: The relative quantity (upper) and decimal logarithm (lower) of newly identified TRs in *T. monococcum* revealed using qPCR and normalized to the *VRN1* reference gene, Figure S2: The relative quantity (upper) and decimal logarithm (lower) of newly identifies tandem repeats in *Ae. speltoides* (SS, candidate donor for B genome) revealed using qPCR and normalized to the *VRN1* reference gene, Figure S3: The relative quantity (upper) and decimal logarithm (lower) of newly identifies tandem repeats in *Ae. tauschii* (DD) revealed using qPCR and normalized to the *VRN1* reference gene, Figure S4: The relative quantity (upper) and decimal logarithm (lower) of newly identifies tandem repeats in *T. durum* (BBAA) revealed using qPCR and normalized to the *VRN1* reference gene, Figure S5: The relative quantity (upper) and decimal logarithm (lower) of newly identifies tandem repeats in *T. aestivum* (BBAADD) revealed using qPCR and normalized to the *VRN1* reference gene, Figure S6: The relative quantity (upper) and decimal logarithm (lower) of newly identifies tandem repeats in *S. cereale* (RR) revealed using qPCR and normalized to the *VRN1* reference gene, Table S1: The efficiency of primers designed on aligned monomer sequences of identified TRs.
