# Supplementary material for: Pipeline for the Rapid Development of Cytogenetic Markers Using Genomic Data of Related Species
Source: Genes (Basel). 2019 Feb 1;10(2):113. doi: 10.3390/genes10020113 (PMC6409974; doi:10.3390/genes10020113)
Supplement: Supplementary file 1 [file genes-10-00113-s001.pdf]

The relative quantity of the TRs and the standard deviations were calculated automatically using LightCycler 96 Software (LightCycler. Relative Quantification. Roche Applied Science Technical Note No. LC 13/2001, available at https://www.gene-quantification.de/roche-rel-quant.pdf)

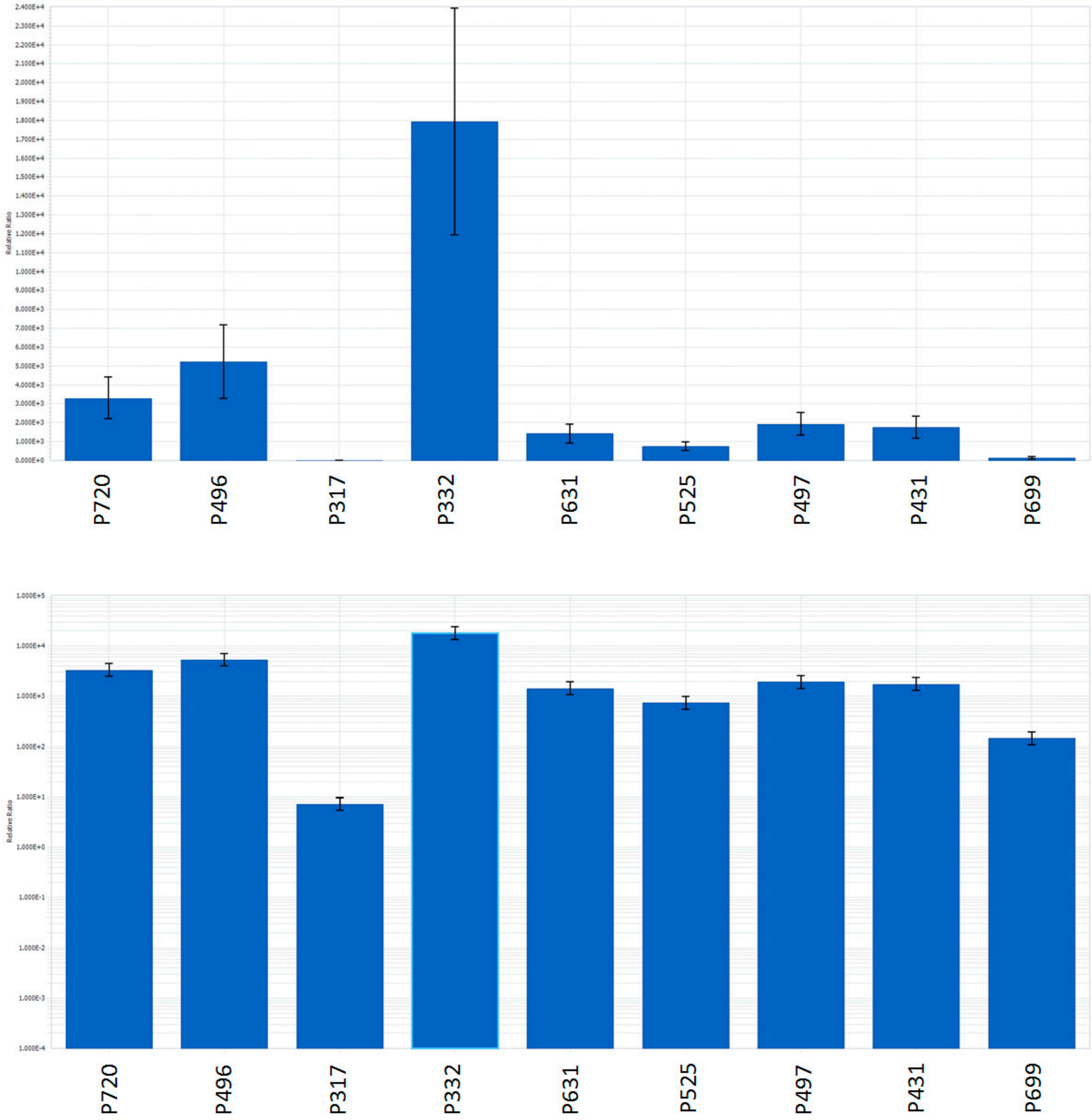

Figure S1. The relative quantity (upper) and decimal logarithm (lower) of newly identified tandem repeats in *Triticum monococcum* (A^mA^m) revealed using qPCR and normalized to the *VRN1* reference gene.

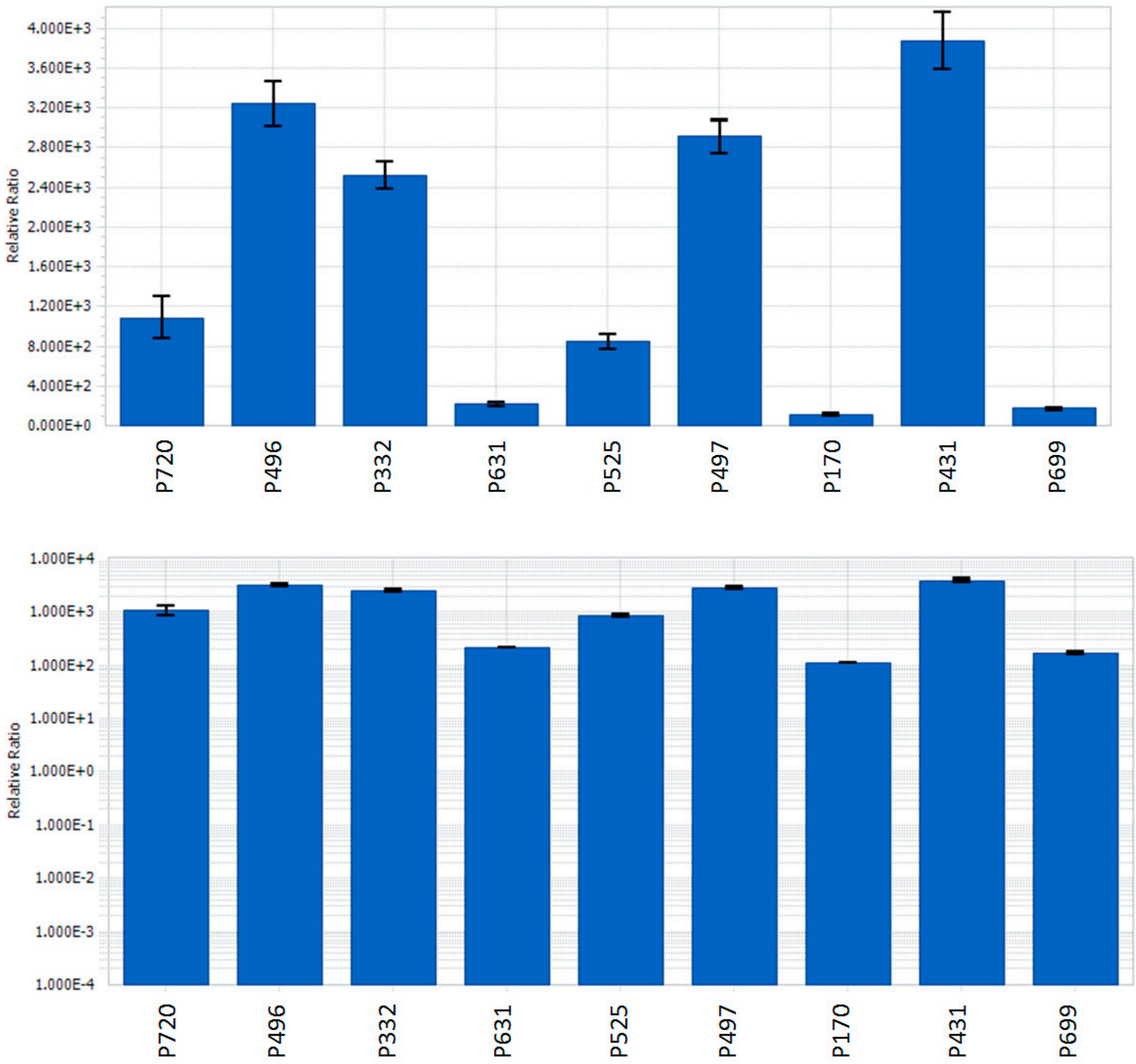

Figure S2. The relative quantity (upper) and decimal logarithm (lower) of newly identified tandem repeats in *Aegilops speltoides* (SS, candidate donor for B genome) revealed using qPCR and normalized to the *VRN1* reference gene.

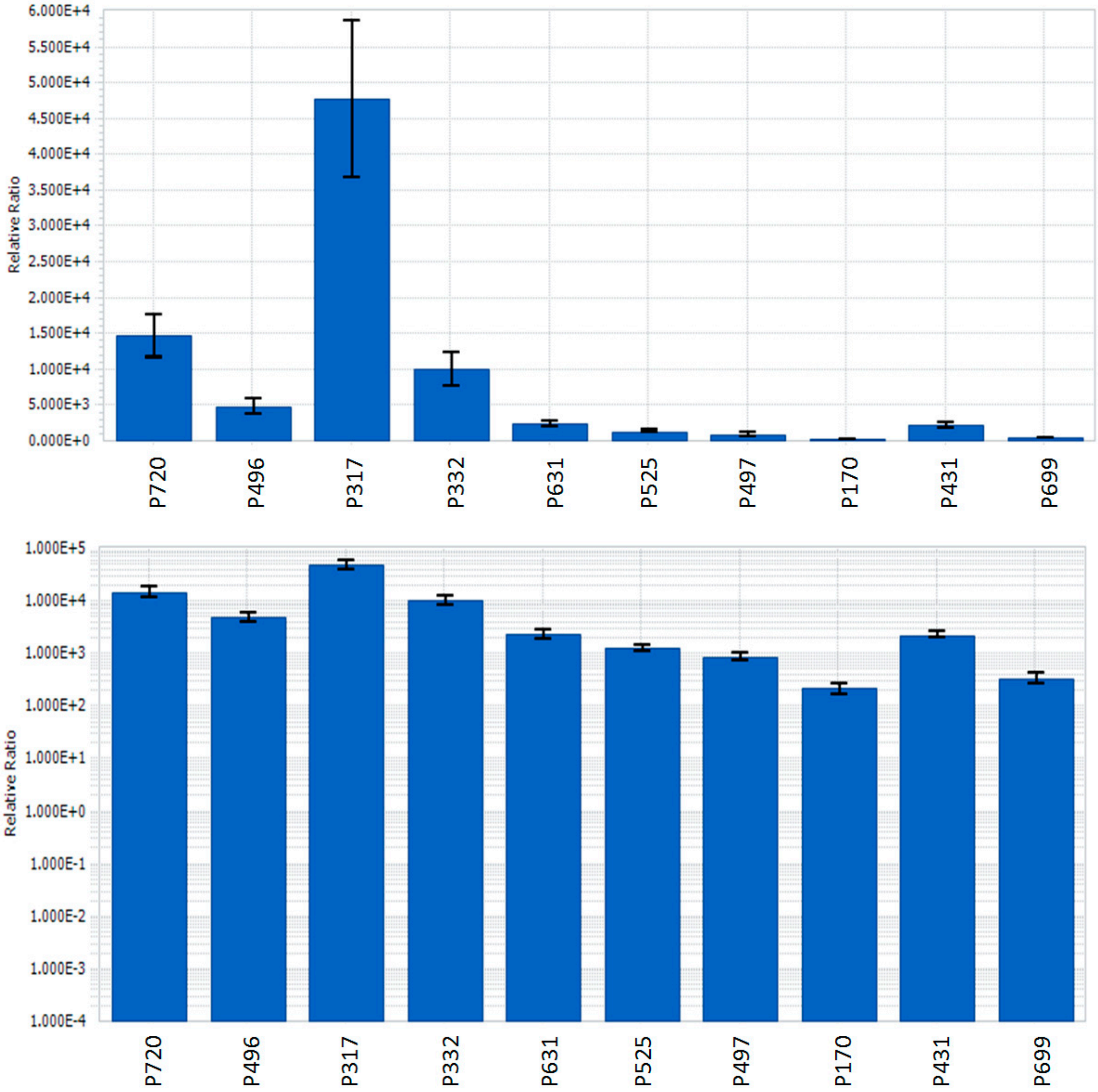

Figure S3. The relative quantity (upper) and decimal logarithm (lower) of newly identified tandem repeats in *Aegilops tauschii* (DD) revealed using qPCR and normalized to the *VRN1* reference gene.

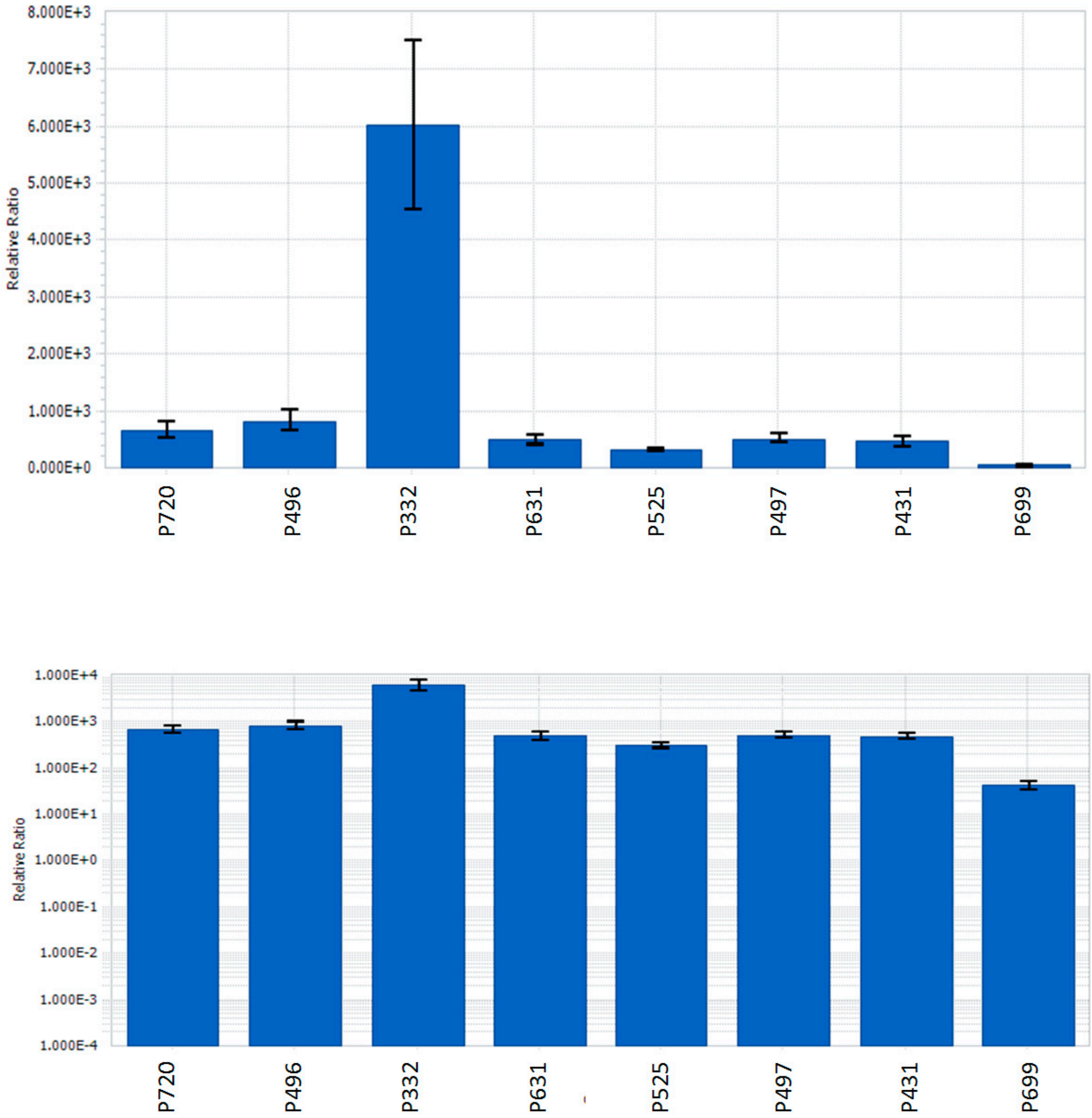

Figure S4. The relative quantity (upper) and decimal logarithm (lower) of newly identified tandem repeats in *Triticum durum* (BBAA) revealed using qPCR and normalized to the *VRN1* reference gene.

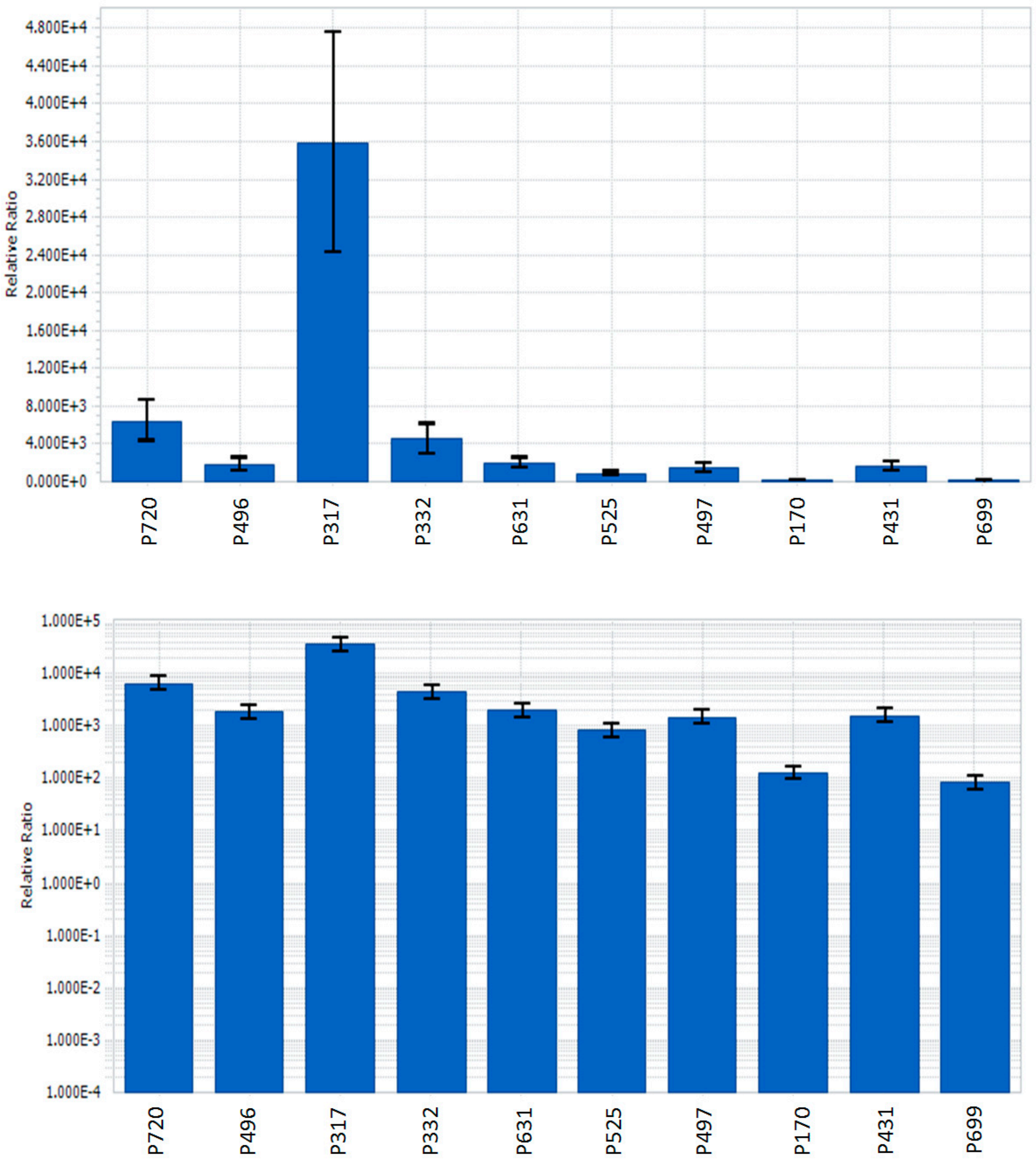

Figure S5. The relative quantity (upper) and decimal logarithm (lower) of newly identified tandem repeats in *Triticum aestivum* (BBAADD) revealed using qPCR and normalized to the *VRN1* reference gene.

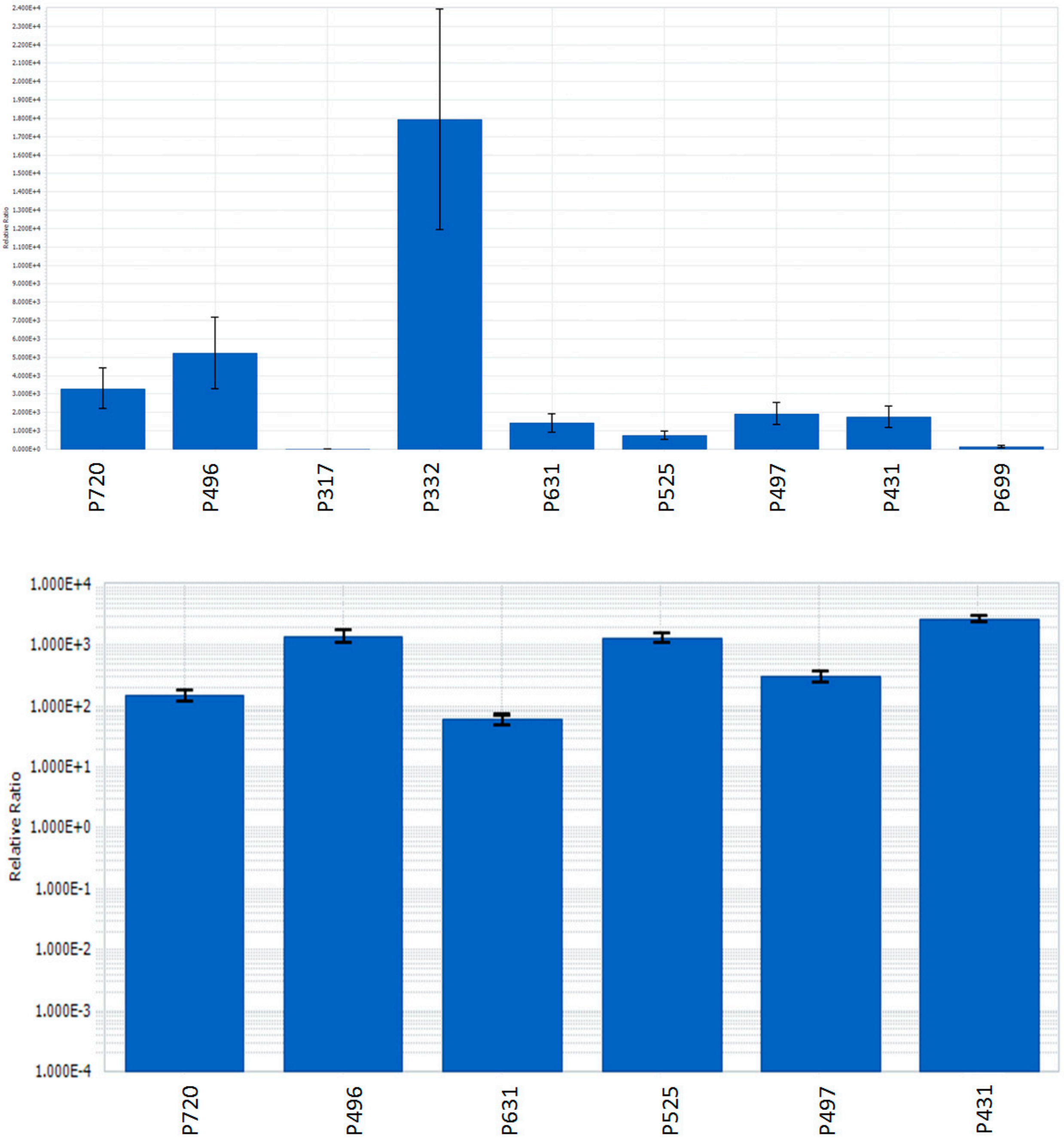

Figure S6. The relative quantity (upper) and decimal logarithm (lower) of newly identified tandem repeats in *Secale cereale* (RR) revealed using qPCR and normalized to the *VRN1* reference gene.
